# Strengthening social belonging through VR-Based Group dance: a controlled trial with community-dwelling older women

**DOI:** 10.3389/fpsyg.2026.1776343

**Published:** 2026-04-14

**Authors:** Yixue Wang, Zhikai Song, Jinjin Zhang

**Affiliations:** 1 Zhengzhou University, Zhengzhou, China; 2 Zhengzhou Technology and Business University, Zhengzhou, China

**Keywords:** group dance, older women, physical activity, social belonging, virtual reality

## Abstract

**Background:**

Social belonging is a critical determinant of psychological well-being and healthy aging, yet it often declines among older women due to social, physical, and contextual barriers. Innovative, engaging, and socially meaningful interventions are needed to address social isolation in this population. Virtual reality (VR)–based group dance has emerged as a promising approach that combines physical activity, social interaction, and immersive experience.

**Methods:**

A randomized controlled trial was conducted with 94 community-dwelling older women aged 60 years and above, who were randomly assigned to an experimental group (VR-based group dance; *n* = 47) or a control group (dose-matched light conventional physical activity; *n* = 47). The intervention lasted 6 weeks, with two sessions per week (20–30 min per session). Perceived social belonging was assessed before and after the intervention using the Sense of Belonging Instrument (SOBI). Analysis of covariance (ANCOVA) was employed to evaluate between-group differences while controlling for baseline scores.

**Results:**

Both groups demonstrated improvements in perceived social belonging; however, the increase was significantly greater in the experimental group compared to the control group. The VR-based group dance intervention showed a substantially larger effect size, indicating that participation in the combined intervention was associated with greater improvements in social belonging relative to the control condition. These findings suggest that while light physical activity provides modest benefits, the integrated VR-based group dance program was associated with stronger social–psychological gains compared with conventional activity.

**Discussion:**

VR-based group dance was associated with meaningful improvements in perceived social belonging among older women. By integrating rhythmic movement, shared group experience, and immersive virtual environments, this approach offers a meaningful social–emotional experience that extends beyond traditional exercise. The findings support the use of VR-based group dance as a complementary strategy to promote social well-being and healthy aging in community-dwelling older adults.

## 1 Introduction

Global population aging is one of the most significant demographic shifts of the 21st century. By 2050, the number of people aged 60 years and older is expected to surpass 2.1 billion—meaning one in every six people worldwide will be an older adult (Dong et al., 2025a; WHO, 2022). This rapid growth has heightened global attention toward supporting psychological well-being, social participation, and quality of life during aging (Bhat et al., 2024). Among these priorities, social belonging—defined as the fundamental human need to feel accepted, valued, and connected within meaningful relationships—has emerged as a critical determinant of healthy aging (Haslam et al., 2018). Older adults with stronger social belonging demonstrate higher life satisfaction, better stress regulation, and lower risks of depression, cognitive decline, and mortality (Hawkley and Cacioppo, 2010; Kim et al., 2025). However, social belonging often declines with age, particularly among older women. Due to a higher likelihood of widowhood, reduced mobility, and fewer opportunities for community engagement, many older women face progressive social withdrawal and emotional vulnerability (Das et al., 2024). Living alone, losing long-term companionship, and cultural gender norms can intensify feelings of loneliness and diminish psychological resilience (Manchana, 2025). This reduction in social connectedness not only contributes to negative emotional states but also limits participation in activities that promote well-being (Slater and Sanchez-Vives, 2016).

Social participation–based interventions, such as group physical activities, are widely recommended to enhance social belonging by fostering shared enjoyment, frequent interpersonal interactions, and a renewed sense of identity (Eime et al., 2013; Li et al., 2024). Nonetheless, many older women remain reluctant to engage in community exercise programs due to fear of falling, transportation difficulties, low confidence, and concerns about judgment from others (Ciaccioni et al., 2021; Wang Z. et al., 2025). These psychological and environmental barriers create a cycle in which limited engagement reinforces social isolation. To break this cycle, innovative and enjoyable interventions are required—especially those that provide emotionally safe environments for social interaction (Faridniya and George, 2025). Virtual Reality (VR) technology has emerged as a promising tool in this area. VR enables immersive, interactive experiences that stimulate positive affect while minimizing real-world risks (Wang L. et al., 2025). Through motion-based gameplay, VR exergames can simulate group exercise in ways that feel joyful, motivating, and socially engaging (Jahanian-Najafabadi et al., 2025). The sense of presence generated in VR supports shared participation even when performed in the same physical space, thus enhancing perceived social relatedness and belonging (Neuhaus et al., 2025).

One particularly appealing format is VR-based group dance, which naturally incorporates rhythm, synchronized movements, laughter, communication, and emotional expression—powerful elements known to strengthen social bonding (Kico et al., 2020). Dance-focused interventions have demonstrated significant effects on psychological well-being, autonomy, body confidence, and group cohesion in older women (Robertson-Wilson et al., 2016). When delivered through VR, group dance may reduce intimidation associated with traditional group settings while maintaining the pleasurable communal experience. In this regard, the theoretical foundation of this study is Fredrickson’s Broaden-and-Build Theory (2001), which asserts that positive emotions broaden individuals’ thought–action repertoires, enabling the development of lasting social and psychological resources (Fredrickson, 2001). VR-based group dance is likely to evoke enjoyment and social enthusiasm—positive emotions that help older women re-connect socially and build resilience against isolation. Therefore, this controlled trial aims to evaluate the effectiveness of a 6-week VR-based group dance program in improving social belonging and broader mental well-being among community-dwelling older women. We hypothesize that participation in VR-based group dance will (1) significantly enhance perceived social belonging by increasing social interaction and shared enjoyment, and (2) contribute to improved emotional well-being compared to a control group. Findings from this study are expected to provide practical guidance for designing accessible, engaging, and socially meaningful interventions to support healthy aging.

## 2 Materials and methods

### 2.1 Research design

This study employed an experimental design in the form of a two-arm, parallel-group randomized controlled trial (RCT) with non-probability convenience sampling. Owing to practical constraints in recruiting community-dwelling older women, participants were recruited voluntarily (Spolarich, 2023) in Wuhan, China through community-based channels. To enhance internal validity and reduce selection bias, eligible participants who completed baseline assessment were randomly allocated in a 1:1 ratio to either an experimental group (VR-based group dance) or a control group (dose-matched conventional physical activity). Randomization was conducted using a computer-generated randomization sequence prepared by a researcher not involved in intervention delivery. Allocation concealment was ensured using sequentially numbered, sealed opaque envelopes opened only after baseline testing was completed.

Because of the nature of the intervention, participant blinding was not feasible. However, outcome assessment was conducted by a researcher not involved in training delivery, and analyses were performed using de-identified group codes.

### 2.2 Participants and recruitment

Participants were community-dwelling older women aged 60 years and older residing in Wuhan, China. Recruitment was conducted through community announcements and senior/community centers. Interested individuals underwent initial screening, followed by in-person baseline assessment and informed consent.

A total of 104 older women initially volunteered and were screened. Ten (10) individuals were excluded prior to completion due to long travel distance, concurrent therapeutic or rehabilitation interventions, voluntary withdrawal for personal reasons, or initiation of other interventions. The final sample comprised 94 participants who completed baseline and post-intervention assessments and adhered to study procedures. Participants were randomly allocated to two equal groups: experimental (*n* = 47) and control (*n* = 47).

*Inclusion criteria were*: (1) women aged ≥ 60 years; (2) living independently in the community; (3) medically cleared to engage in light-to-moderate physical activity; (4) ability to understand study instructions and complete self-report questionnaires; (5) no known contraindications to VR exposure (e.g., epilepsy or marked photosensitivity); and (6) provision of written informed consent.

*Exclusion criteria included*: (1) unstable cardiovascular, neurological, or musculoskeletal conditions contraindicating participation; (2) severe balance impairment posing unacceptable safety risk; (3) history of epilepsy or significant photosensitivity; (4) severe uncorrected sensory impairment preventing safe participation; (5) concurrent enrollment in other structured exercise/psychosocial programs likely to confound outcomes; and (6) withdrawal of consent at any stage.

Participant demographic characteristics are presented in Table 1.

**Table 1:** Demographic characteristics of participants.

Variable	Control group (*n* = 47)	Experimental group (*n* = 47)	Total (*N* = 94)
Age (years), mean ± SD	70.9 ± 5.2	70.4 ± 5.5	70.6 ± 5.3
Age range (years)	60–83	60–82	60–83
Marital status, *n* (%)			
Married	19 (40.4%)	18 (38.3%)	37 (39.4%)
Widowed	22 (46.8%)	24 (51.1%)	46 (48.9%)
Divorced/single	6 (12.8%)	5 (10.6%)	11 (11.7%)
Living arrangement, *n* (%)			
Living alone	20 (42.6%)	22 (46.8%)	42 (44.7%)
Living with family	27 (57.4%)	25 (53.2%)	52 (55.3%)

### 2.3 Data collection tools

#### 2.3.1 Primary outcome: social belonging

Assessments were conducted at two time points: pre-intervention (baseline) and post-intervention (end of week 6). Demographic and health-related information (age, education, marital status, living arrangement, comorbidities, and medication status) was collected at baseline. In this regard, perceived social belonging was assessed using the Sense of Belonging Instrument (SOBI) developed by Hagerty and Patusky (1995). The SOBI is a 27-item self-report instrument comprising two subscales: Psychological Sense of Belonging (SOBI-P; 18 items) and Antecedents to Sense of Belonging (SOBI-A; 9 items) (Hagerty and Patusky, 1995). Items are rated on a 4-point Likert scale, with higher scores indicating a stronger sense of social belonging. The SOBI was scored according to the original scoring guidelines, including reverse scoring where applicable. Scale reliability in the present sample was evaluated using Cronbach’s alpha.

#### 2.3.2 Safety monitoring

Participants in the VR group were monitored for VR-related discomfort throughout the program. A brief cybersickness screening questionnaire was administered after each VR session, and any adverse events (e.g., dizziness, nausea, headache, imbalance) were documented. Participants were permitted to pause or discontinue sessions at any time, and standardized stop-rules were applied to ensure safety.

### 2.4 Data analysis procedure

Statistical analyses were performed using IBM SPSS Statistics (version 26). Descriptive statistics (means, standard deviations, and frequencies) were calculated for all study variables. Baseline group equivalence was examined using independent-samples t-tests for continuous variables and chi-square tests for categorical variables.

Normality was assessed using the Kolmogorov–Smirnov test, and homogeneity of variances was evaluated using Levene’s test. The primary intervention effect on social belonging was tested using analysis of covariance (ANCOVA), with the post-test SOBI total score as the dependent variable, group as the fixed factor, and baseline SOBI total score as the covariate. Additional ANCOVAs were conducted for the SOBI subscales (SOBI-P and SOBI-A) to examine differential effects across belonging dimensions. Statistical significance was set at *p* < 0.05 (two-tailed), and effect sizes were reported to support practical interpretation. Because the final sample (*N* = 94) provided complete baseline and post-test data, analyses were conducted on the complete dataset.

### 2.5 Implementation process of the training protocol for experimental and control groups

An interdisciplinary team with expertise in exercise science, geriatric health, and VR-based training developed and implemented the protocol to ensure safety and feasibility for older adults. Prior to the intervention, participants attended an orientation session in which study procedures were explained, safety instructions were provided, and participants were familiarized with the training environment. Written informed consent was obtained from all participants, attendance was recorded at each session, and adherence was monitored throughout the trial.

#### 2.5.1 Experimental group: VR-based group dance program

Participants in the experimental group completed a VR-based group dance program over 6 weeks, with two sessions per week for a total of 12 sessions. Each session lasted approximately 20–30 min, including brief warm-up and cool-down periods. The intervention was delivered using a standalone, commercially available head-mounted VR display with six-degrees-of-freedom tracking and an interactive dance-based application that provided rhythm-guided movement cues and real-time feedback. Sessions were conducted in small groups under supervision to promote safety and to support shared engagement.

The program progressed from simpler rhythmic routines to gradually more complex movement sequences across the 6-week period while maintaining light-to-moderate exertion appropriate for older adults. Standardized session procedures and instructor prompts were used to ensure intervention fidelity.

#### 2.5.2 Control group: dose-matched conventional physical activity

Participants in the control group completed a dose-matched conventional physical activity program over the same 6-week period, with the same frequency (two sessions per week), total number of sessions (12 sessions), and duration (20–30 min per session). Sessions consisted of mild walking combined with very light exercises appropriate for older adults. Each session included a short warm-up, gentle walking at a comfortable pace, and low-intensity mobility/stretching exercises, followed by a brief cool-down. The control condition was designed to match time and general activity exposure while excluding VR immersion and VR-specific interactive features.

## 3 Results

### 3.1 Participant flow

**Figure 1:**
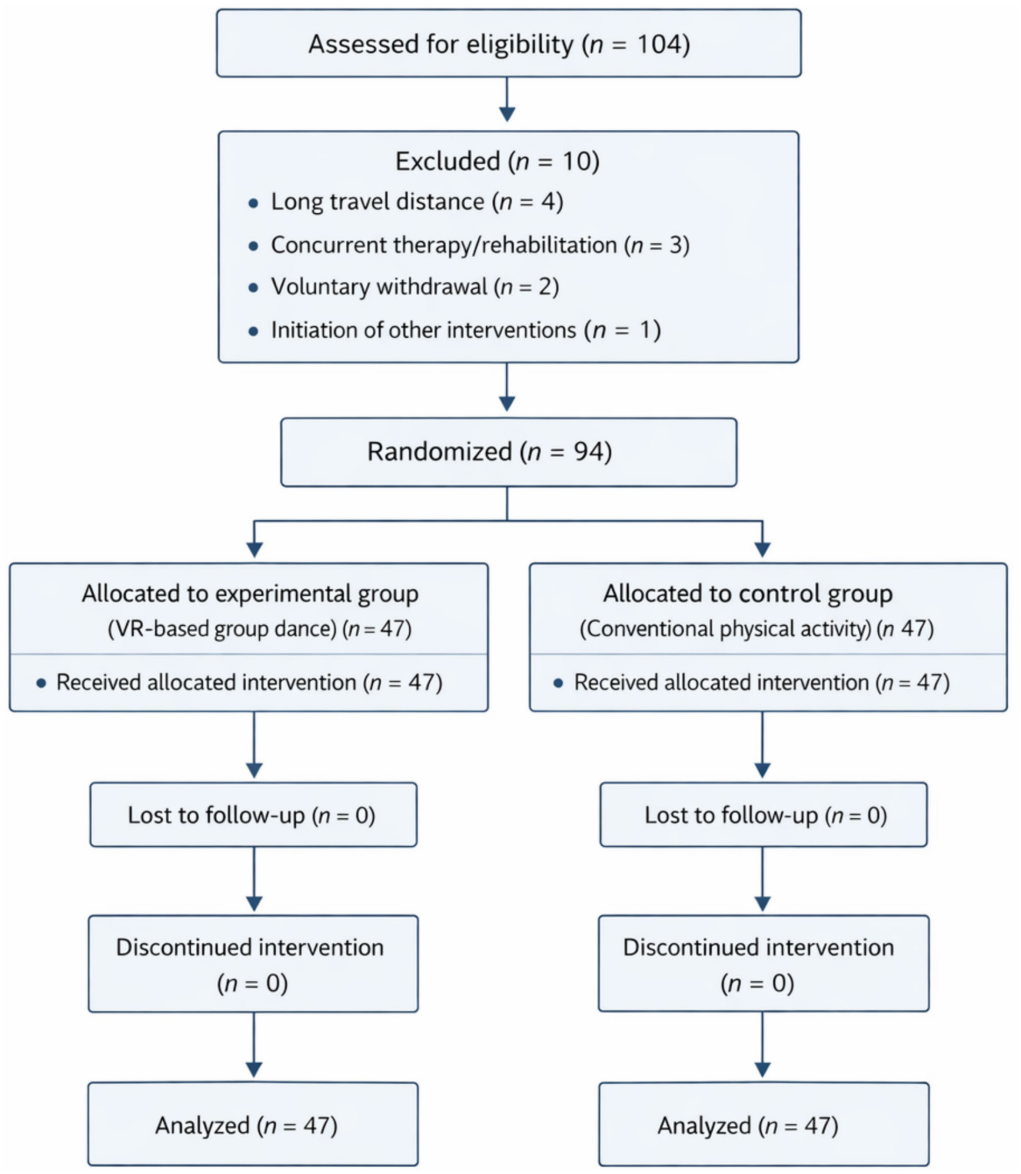
CONSORT flow diagram of participant recruitment, randomization, follow-up, and analysis.

A total of 104 older women initially volunteered and were assessed for eligibility. Ten participants were excluded prior to randomization due to long travel distance (*n* = 4), concurrent therapeutic or rehabilitation interventions (*n* = 3), voluntary withdrawal (*n* = 2), or initiation of other interventions (*n* = 1). The remaining 94 eligible participants were randomly allocated in a 1:1 ratio to the experimental group (VR-based group dance; *n* = 47) or the control group (dose-matched conventional physical activity; *n* = 47). All randomized participants completed the 6-week intervention and post-intervention assessment. Therefore, no participants were lost to follow-up, and all participants were included in the final analysis (Figure 1).

### 3.2 Assumption testing

As shown in Table 2, the Kolmogorov–Smirnov test indicated that SOBI total scores at baseline and post-intervention were approximately normally distributed within both groups (all *p* > 0.05). Levene’s test further supported the assumption of homogeneity of variances for post-test SOBI scores (*p* > 0.05) (Table 3). In addition, the homogeneity of regression slopes assumption for ANCOVA was examined by testing the interaction between baseline SOBI and group; the interaction was not significant (*p* > 0.05), supporting the appropriateness of ANCOVA.

**Table 2:** Kolmogorov–Smirnov test for normality (SOBI total).

Group	Variable	*N*	Mean	Std. deviation	Test statistic	Asymp. sig. (2-tailed)
Control	Pre_SOBI	47	66.40	7.10	0.090	0.200
Control	Post_SOBI	47	68.00	7.20	0.085	0.200
Experimental	Pre_SOBI	47	65.80	7.40	0.094	0.200
Experimental	Post_SOBI	47	75.20	7.00	0.079	0.200

SOBI ranges from 27 to 108; higher scores indicate stronger sense of social belonging.

**Table 3:** Levene’s test for equality of error variances (Post_SOBI).

F	df1	df2	Sig.
0.680	1	92	0.411

### 3.3 Descriptive results

Table 4 presents descriptive statistics for SOBI total scores. Both groups showed a positive change from baseline to post-test. However, the magnitude of improvement was notably larger in the experimental group following the VR-based group dance program (Δ = +9.40) compared with the control group, which participated in dose-matched mild walking and very light exercises (Δ = +1.60).

**Table 4:** Descriptive indicators of SOBI total (pre- and post-test).

Group	Time	*N*	Minimum	Maximum	Mean	Std. Deviation	Variance
Control	Pre_SOBI	47	52	82	66.40	7.10	50.41
Control	Post_SOBI	47	54	85	68.00	7.20	51.84
Experimental	Pre_SOBI	47	50	83	65.80	7.40	54.76
Experimental	Post_SOBI	47	60	92	75.20	7.00	49.00

### 3.4 Homogeneity of variance

As shown in Table 3, Levene’s test confirmed that the variance of post-test SOBI scores was statistically equivalent across groups (*p* = 0.411), satisfying the homogeneity assumption for ANCOVA.

### 3.5 Within-group changes

To demonstrate that the control group also experienced a small positive improvement, paired-samples t-tests were conducted within each group. The control group showed a modest but positive pre–post increase in SOBI [*t*(46) = 2.13, *p* = 0.038], corresponding to a small effect (partial *η*^2^ = 0.090). In contrast, the experimental group showed a substantially larger improvement [*t*(46) = 8.23, *p* < 0.001], corresponding to a large effect (partial *η*^2^ = 0.595).

### 3.6 Primary outcome analysis (ANCOVA)

An ANCOVA was conducted to compare post-intervention SOBI total scores between groups while controlling for baseline SOBI. As shown in Table 5, the adjusted group effect was statistically significant and large: *F*(1,91) = 58.00, *p* < 0.001, partial *η*^2^ = 0.389. This indicates that approximately 38.9% of the variance in post-test sense of belonging was attributable to group assignment beyond baseline belonging, supporting the effectiveness of the VR-based group dance intervention. Although group assignment accounted for a substantial proportion of variance in post-test social belonging scores (partial η^2^ = 0.389), this statistical effect reflects the overall impact of the combined VR-based group dance intervention rather than the isolated effect of virtual reality alone. Given the multicomponent nature of the experimental condition (immersive technology + dance-specific movement + group interaction), the present design does not permit identification of which specific element(s) were responsible for the observed change.

**Table 5:** ANCOVA results for SOBI total.

Source	Type III sum of squares	*df*	Mean square	*F*	Sig.	Partial eta squared
Corrected model	1960.000	2	980.000	35.000	<0.001	0.435
Intercept	712.000	1	712.000	25.429	<0.001	0.218
Pre_SOBI	336.000	1	336.000	12.000	0.001	0.117
Group	1624.000	1	1624.000	58.000	<0.001	0.389
Error	2548.000	91	28.000	—	—	—
Corrected total	4508.000	93	—	—	—	—

### 3.7 VR safety and adverse events

Throughout the intervention period, no serious adverse events were reported in the VR-based group dance program. A small number of participants reported mild and transient symptoms such as brief dizziness or visual discomfort during initial sessions; however, these symptoms resolved quickly without requiring medical attention or withdrawal from the study. No participants discontinued the intervention due to VR-related discomfort.

**Figure 2:**
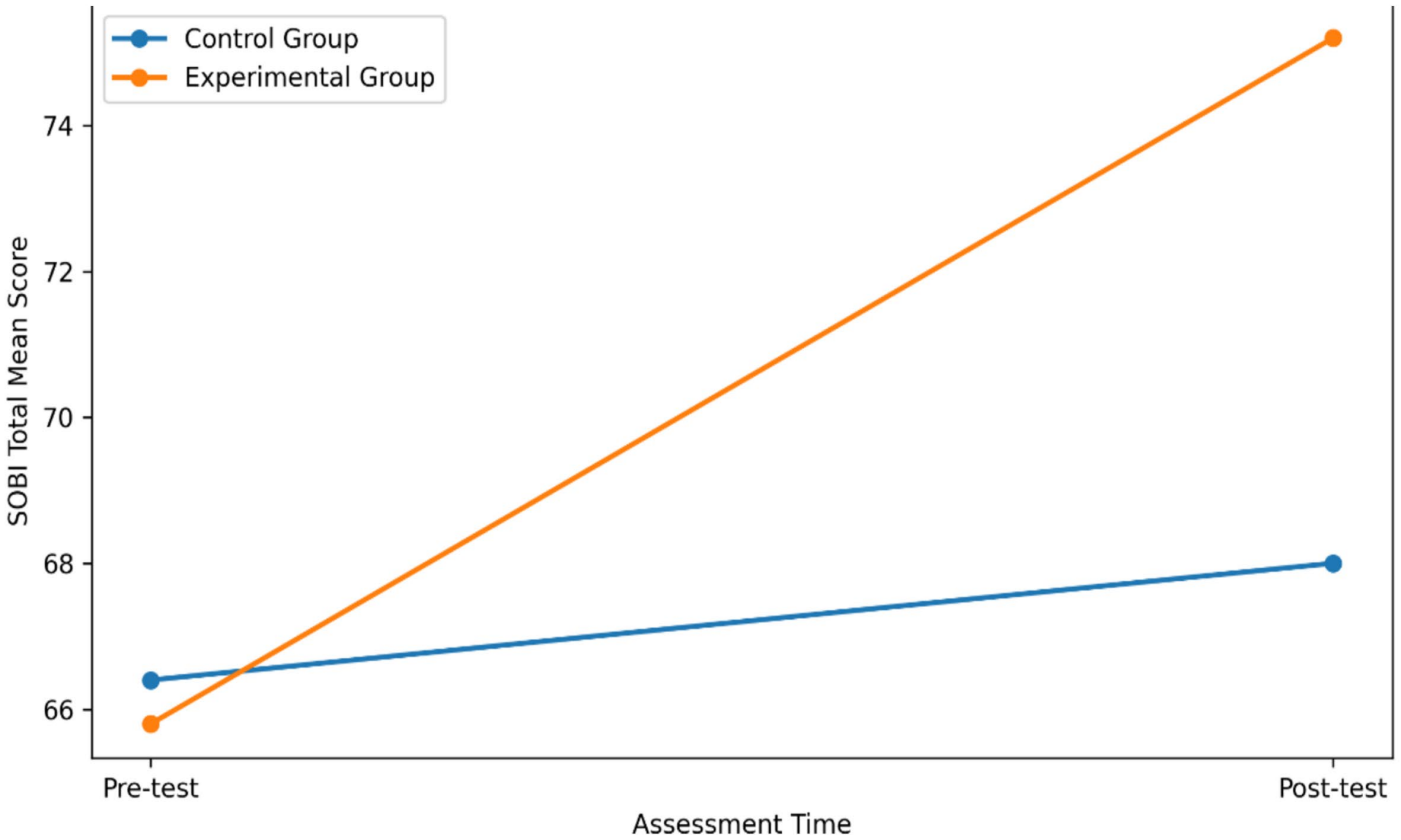
Mean changes in social belonging across groups.

To facilitate a clearer understanding of the observed changes, Figure 2 presents the mean pre- and post-test SOBI scores for both the experimental and control groups.

## 4 Discussion

The findings of the present study indicate that participation in a virtual reality–based group dance intervention was associated with a significant increase in perceived social belonging among community-dwelling older women. Although the control group—who engaged in light physical activities such as mild walking and very low-intensity exercises—also demonstrated a modest improvement in social belonging, the magnitude of this effect was substantially smaller than that observed in the experimental group. This pattern of results suggests that while physical activity alone may provide a limited protective effect against social isolation in later life, more substantial and enduring changes in social–psychological constructs are likely to require richer, experience-based interventions. This finding is consistent with a broad body of research identifying physical activity as an effective non-pharmacological intervention for enhancing mental health and quality of life in older adulthood (Bauman et al., 2016; Dong et al., 2025b; Netz et al., 2005). Numerous studies have shown that even simple forms of exercise, such as regular walking, can reduce depressive symptoms, anxiety, and feelings of loneliness among older (Lim et al., 2023; Mendes et al., 2016). Accordingly, the modest improvement observed in the control group in the present study may be attributed to these well-documented mechanisms, particularly increased minimal social contact, greater temporal structure in daily routines, and physiological activation associated with bodily movement.

However, the key finding of the present study lies in the significantly greater improvement observed in the experimental group, highlighting the added value of the combined VR-based group dance intervention (i.e., immersive technology integrated with dance-based, group-level activity). An increasing body of research indicates that VR-based group dance intervention—particularly when combined with rhythmic or interactive movement—can yield stronger psychological and social outcomes than traditional exercise programs (Kim and Choi, 2025; Wang L. et al., 2025). Immersive virtual environments may facilitate a sense of social presence and interpersonal synchrony, which have been suggested in prior literature as factors associated with the development of social belonging (Moonen et al., 2024; Wei et al., 2026). In line with this evidence, Dong et al. (2025a, 2025b) reported that older adults participating in VR-based exercise programs experienced improvements in future-oriented psychological resources, such as hope for life, which are conceptually related to social belonging and may share similar affective pathways. Although the primary outcome in their study was hope for life, from a theoretical perspective, future-oriented hope and social belonging represent closely related protective psychological constructs in later life, both of which may be influenced through similar affective and social pathways activated by immersive VR interventions (Dong et al., 2025b). These findings suggest that immersive VR environments can lower psychological barriers to participation and create a safer context for engagement, thereby fostering positive psychosocial outcomes—a mechanism that likely contributed to the enhancement of social belonging among older women in the present study as well (Wang L. et al., 2025). Further supporting this interpretation, Faridniya and George (2025) found that gamified VR-based exercise interventions play a substantial role in enhancing participation, inclusivity, and psychological well-being. These findings align with the present study, suggesting that integrating technology, physical activity, and interactive elements can transform exercise into a meaningful social–emotional experience associated with enhanced social belonging (Faridniya and George, 2025). Moreover, recent research has shown that VR-based exercise interventions can be effective in promoting mental health and preventing emotional disorders among vulnerable populations. Although the target groups in these studies differed from the current sample, the convergence of findings suggests that VR-based group dance intervention—by strengthening emotional regulation, perceived control, and meaningful engagement—may exert similar protective effects in older women, as reflected in the observed improvement in social belonging in the present study (Wei et al., 2026).

From a theoretical perspective, the findings of the present study can be effectively interpreted within Fredrickson’s Broaden-and-Build Theory. This framework posits that positive emotions broaden individuals’ momentary thought–action repertoires and, over time, contribute to the development of enduring psychological and social resources (Fredrickson, 2001; Roth et al., 2024). Participation in VR-based group dance may have elicited a range of positive emotions, such as enjoyment and social enthusiasm, which could plausibly contribute to enhanced social interaction and, in turn, greater perceived social belonging. In this context, dance as a mind–body activity appears to have a greater capacity to evoke positive emotions and social engagement than many forms of traditional exercise (Trotta, 2025; Worthen-Chaudhari et al., 2025). Prior research has shown that movement synchrony—a defining feature of group dance-can enhance social bonding, trust, and group cohesion (Dieterich-Hartwell, 2025; Suberry and Bodner, 2024; Tarr et al., 2014). When such synchrony occurs within a shared virtual environment, its effects may be further amplified, as suggested by previous research, and could represent a plausible mechanism underlying the observed improvements in social belonging (Pan and Hamilton, 2018). Moreover, virtual reality may help reduce common psychological barriers to social participation among older women. Studies indicate that older women are more likely than men to experience concerns related to social evaluation, diminished bodily confidence, and anxiety about social comparison (Griffin and Phoenix, 2015; Swami et al., 2021). By reducing direct visibility and providing a psychologically safe environment, virtual settings may facilitate more active participation (Wang Z. et al., 2025). This feature is particularly important for women who feel uncomfortable in traditional exercise contexts and may contribute to enhanced experiences of social acceptance and perceived value—two core components of social belonging (Hagerty et al., 1992; Haslam et al., 2018). The findings of the present study are also consistent with previous research indicating that VR-based group dance intervention can enhance intrinsic motivation and enjoyment of physical activity—factors that are indirectly associated with social belonging (Farič et al., 2019; Zhao et al., 2020). Increased motivation and enjoyment may facilitate more sustained engagement in group-based activities and create greater opportunities for meaningful social interaction. At the same time, it is important to consider that part of the observed improvement may be influenced by a novelty effect associated with immersive virtual reality technology. For participants with limited prior exposure to VR, the innovative and stimulating nature of the technology may have generated heightened enthusiasm, curiosity, or short-term engagement that positively influenced subjective evaluations of social experience. In this sense, some portion of the increased social belonging scores may reflect initial excitement related to technological novelty rather than fully stabilized psychosocial change. Nevertheless, even if novelty contributed to the magnitude of the effect, it may still represent a meaningful entry point for facilitating social engagement among older adults, particularly those who might otherwise be reluctant to participate in conventional group activities. Future longitudinal research is needed to determine whether these benefits persist beyond the initial exposure phase.

These findings may serve as applied evidence for the design and implementation of social health promotion programs for older adults, particularly within community-based settings, senior health centers, and primary mental health prevention initiatives. From a practical perspective, integrating virtual reality technology with movement-based group activities may represent an effective strategy for increasing social participation among older women—a population that often faces greater barriers to engagement in collective activities. Accordingly, VR-based group dance intervention may be employed as a complementary approach to traditional exercise programs, offering a more engaging and meaningful experience that simultaneously enhances adherence to physical activity and social interaction. It should be noted that these proposed psychological processes are inferred from existing theoretical frameworks and prior empirical evidence; however, they were not directly measured in the present study and should therefore be interpreted as plausible explanatory mechanisms rather than confirmed causal pathways. Moreover, such interventions may assist policymakers in the field of healthy aging and designers of psychological interventions in adopting low-risk, technology-assisted strategies to reduce social isolation and strengthen social belonging among community-dwelling older adults, without the need for costly resources or invasive clinical interventions.

## 5 Limitations and directions for future research

Several limitations should be acknowledged. First, although a randomized controlled design was employed, the intervention combined virtual reality immersion, dance-based movement, and group interaction within a single program. Therefore, the findings reflect the effectiveness of the combined VR-based group dance intervention rather than the isolated effect of VR itself. Future multi-arm studies are needed to disentangle the unique contributions of technological immersion and activity type. Second, social belonging was assessed solely through self-report (SOBI), which may be influenced by social desirability, expectancy effects, and potential novelty-related enthusiasm associated with immersive technology. The absence of behavioral or relational indicators limits interpretability. Future research should incorporate multi-method assessments. Third, pre-existing social conditions (e.g., chronic loneliness, prior community engagement, or familiarity among participants) were not fully controlled and may have influenced responsiveness to the intervention. Including key social indicators or stratified randomization procedures in future studies would strengthen internal validity. Fourth, the use of convenience sampling and the inclusion of only community-dwelling older women from a single city limit generalizability. Broader and probability-based sampling strategies are recommended. Finally, the relatively short intervention period and lack of follow-up assessment prevent conclusions about the durability of effects. Longitudinal studies are necessary to evaluate the sustainability of improvements in social belonging.

## 6 Conclusion

The findings of this randomized controlled trial indicate that while light and regular physical activity can lead to modest improvements in perceived social belonging among community-dwelling older women, a combined VR-based group dance intervention was substantially more effective in strengthening this psychological construct. By integrating rhythmic movement, shared group experience, and an immersive virtual environment, this approach appears to create favorable conditions for enhancing social interaction, positive emotions, and a sense of social connectedness. Importantly, the observed benefits should be interpreted as the effect of the integrated intervention as a whole, rather than being attributed to virtual reality alone. Overall, VR-based group dance intervention may represent a valuable and complementary strategy for promoting social belonging and supporting healthy aging among older adults.

## Data Availability

The original contributions presented in the study are included in the article/supplementary material, further inquiries can be directed to the corresponding author.
